# Ultra-Rare BRD9 Loss-of-Function Variants Limit the Antiviral Action of Interferon

**DOI:** 10.1038/s41598-022-19648-w

**Published:** 2022-09-13

**Authors:** Nina K. Mair, Benjamin G. Hale

**Affiliations:** 1 Institute of Medical Virology, University of Zurich, 8057 Zurich, Switzerland; 2 Life Science Zurich Graduate School, ETH and University of Zurich, 8057 Zurich, Switzerland

**Keywords:** Innate immunity, Cytokines, Interferons, Infectious diseases, Viral infection, Infection, Epigenetics, Virology, Viral host response

## Abstract

The human type I interferon (IFN) system is central to innate immune defense, and is essential to protect individuals against severe viral disease. Consequently, genetic disruption of IFN signaling or effector mechanisms is extremely rare, as affected individuals typically suffer life-threatening infections at an early age. While loss-of-function (LOF) mutations in canonical JAK-STAT signaling genes (such as IFNAR2, TYK2, STAT1, STAT2 and IRF9) have previously been characterized, little is known about the consequences of mutations in other human factors required for IFN signaling. Here, we studied the impact of rare human genetic variants in the recently identified contributor to IFN-stimulated gene expression and antiviral activity, bromodomain-containing protein 9 (BRD9). Using a cell-based BRD9 knock-out and reconstitution model system, we functionally assessed 12 rare human BRD9 missense variants predicted to impair protein function, as well as 3 ultra-rare human BRD9 LOF variants that lead to truncated versions of BRD9. As compared to wild-type BRD9, none of the 12 BRD9 missense variants affected the ability of exogenous IFN to limit virus replication. In contrast, all 3 truncated BRD9 LOF variants failed to allow exogenous IFN to function efficiently, as evidenced by exacerbated replication of an IFN-sensitive virus and diminished IFN-stimulated gene expression. Thus, while no homozygous BRD9 LOF carriers have yet been identified, our results predict that such extremely rare individuals would exhibit a compromised ability to mount a fully protective IFN-mediated antiviral response. Genetic variation in BRD9 could be considered in future studies to understand the infection susceptibility of some individuals.

## Introduction

The human type I interferon (IFNα/ß) system is central to host antiviral defenses and is a critical part of innate immunity. At a simplified level, cells can detect that they are infected via host-encoded sensor proteins, such as RIG-I, mda-5 or cGAS, which activate intracellular signaling cascades leading to the production and secretion of IFN cytokines. Type I IFNs bind to the heterodimeric IFNAR1/IFNAR2 receptor complex expressed on the surface of most cells, and trigger a JAK-STAT signaling response canonically involving TYK2, JAK1, STAT1, STAT2 and IRF9^1^. The activated STAT1-STAT2-IRF9 transcription factor complex then stimulates the transcription and translation of hundreds of IFN-stimulated genes (ISGs), which together act to limit virus replication and disease severity through multiple mechanisms^2^. Critically, congenital errors of either IFN signaling pathway components or ISGs impair innate antiviral control mechanisms, and can result in increased fatal susceptibility to a spectrum of viral diseases and innocuous live-attenuated viral vaccines^3,4^. For example, loss-of-function mutations in genes such as *IFNAR2*, *TYK2*, *STAT1*, *STAT2* and *IRF9* have been identified in several young individuals who have suffered severe infections with common viruses^5–9^. Furthermore, human variants in antiviral ISG effector proteins, including MxA, OAS1 and IFITM3, have been shown to reduce the antiviral control of emerging pandemic viruses, specifically influenza (MxA and IFITM3) or SARS-CoV-2 (OAS1)^10–12^.

Typically, identification of susceptibility-associated host genetic variants in the IFN system has been achieved through the whole-genome or whole-exome sequencing of rare individual patients with otherwise unexplained severe viral infections, and downstream analyses are focused on predicting loss-of-function missense variants in known IFN pathway genes^3^. Alternatively, large genome-wide association studies can be performed at a cohort level when the number of individuals affected by severe infection is greater, as for example during infections with an antigenically novel pandemic virus^13,14^. In this study we adopted a third approach based on unbiased functional screening of human genetic variants reported in the Genome Aggregation Database (gnomAD), a publicly-available resource comprising over 125,000 human exome and 15,000 human genome sequences, which are not necessarily associated with any known disease^15^. Our rationale was that loss-of-function missense genetic variants may exist in human populations and have the potential to compromise IFN-mediated antiviral immunity, but have not yet been identified due to their extreme rarity or recessive nature, or due to their existence in non-canonical IFN signaling pathway components that are not part of most targeted analyses. In this latter regard, we therefore focused our attention on bromodomain-containing protein 9 (BRD9), an epigenetic reader and defining subunit of non-canonical BAF (ncBAF) chromatin remodeling complexes, that we and others have newly characterized as a cellular contributor to efficient ISG expression and antiviral immunity^16–18^. We now explore a range of rare human *BRD9* genetic variants that are predicted to be deleterious in human genomes, and use functional reconstitution experiments in *BRD9* knock-out cells to assess their impact on IFN-mediated antiviral activity. Notably, we identify three naturally occurring, ultra-rare, human *BRD9* loss-of-function variants that significantly limit the cell’s ability to mount an effective antiviral IFN response.

## Results and discussion

### A *BRD9* knock-out cell-line as a tool to investigate the antiviral function of missense variants

**Figure 1:**
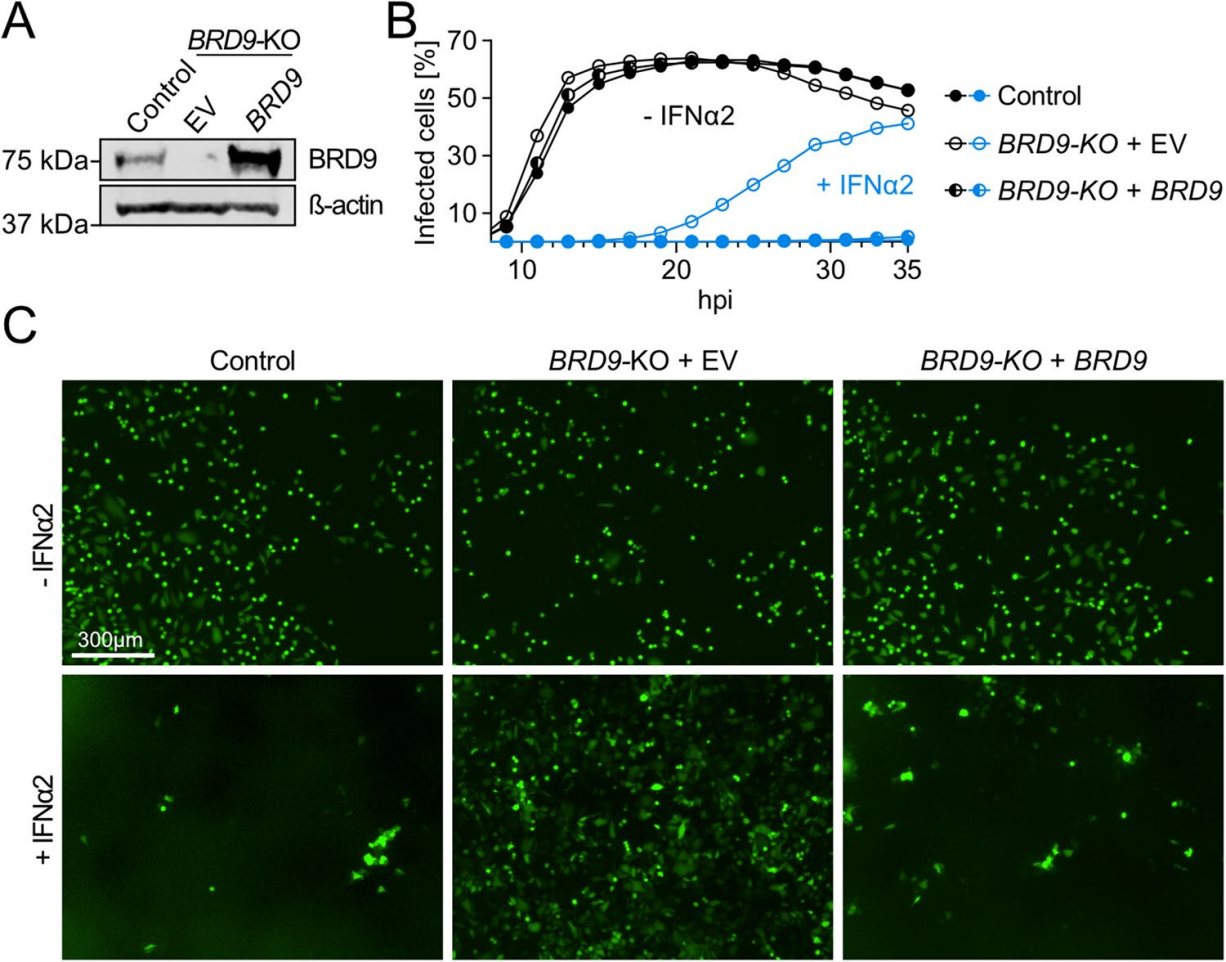
Characterization of a *BRD9* knock-out cell-line. (**A**) Western blot analysis of lysates from a non-edited control A549 cell-line and a *BRD9* knock-out (KO) A549 cell-line that had been stably-transduced with lentiviruses expressing BRD9 or an empty vector (EV) control. BRD9 and β-actin were detected with specific antibodies. (**B**) Cells described in (**A**) were stimulated with 0 or 1,000 IU/mL of IFNα2 for 16 h prior to infection with VSV-GFP at an MOI of 1 PFU/cell. The percentage of infected cells was quantified via GFP signal measurements and cell confluence over a 35 h period. Data shown are representative of three biologically independent experiments. (**C**) Representative fluorescent images of experiments described in (**B**). Scale bar represents 300 μm. Source data underlying the western blots in this figure can be found in the Supplementary Information file.

Our previous work demonstrated that complete genetic knock-out of human *BRD9*, or small molecule triggered degradation of the BRD9 protein, compromises efficient IFN-stimulated gene expression and antiviral activity^16^. As an experimental tool to dissect the impact of naturally occurring human *BRD9* missense variants, we re-validated our previously described CRISPR/Cas9-generated A549-based *BRD9* knockout (KO) cell-line in comparison to a non-edited control^16^. Western blot analysis confirmed the lack of BRD9 protein expression in the KO cell-line, and validated that BRD9 levels could be restored by stable transduction with a lentiviral vector encoding wild-type human *BRD9* (Fig. 1A). Furthermore, while vesicular stomatitis virus (VSV-GFP; an IFN-sensitive model virus) replicated similarly in both non-edited control cells and *BRD9*-KO cells under baseline conditions, VSV-GFP was highly attenuated for replication in IFNα2-treated non-edited control cells, but not in *BRD9*-KO cells (Fig. 1B,C). This is consistent with the specific inability of *BRD9*-KO cells to mount an efficient antiviral ISG response^16^. Importantly, *BRD9*-KO cells reconstituted with wild-type human BRD9 were fully able to mount an IFNα2-mediated antiviral ISG response against VSV-GFP (Fig. 1B,C), confirming that the defect in *BRD9*-KO cells is specific to lack of BRD9. These data validate the *BRD9*-KO cell-line, with reconstitution of *BRD9*, as a tractable experimental system to assess the antiviral function of cloned *BRD9* missense variants.

### Rarity of *BRD9* missense variants in human genomes suggests the importance of *BRD9* sequence conservation in vivo

**Figure 2:**
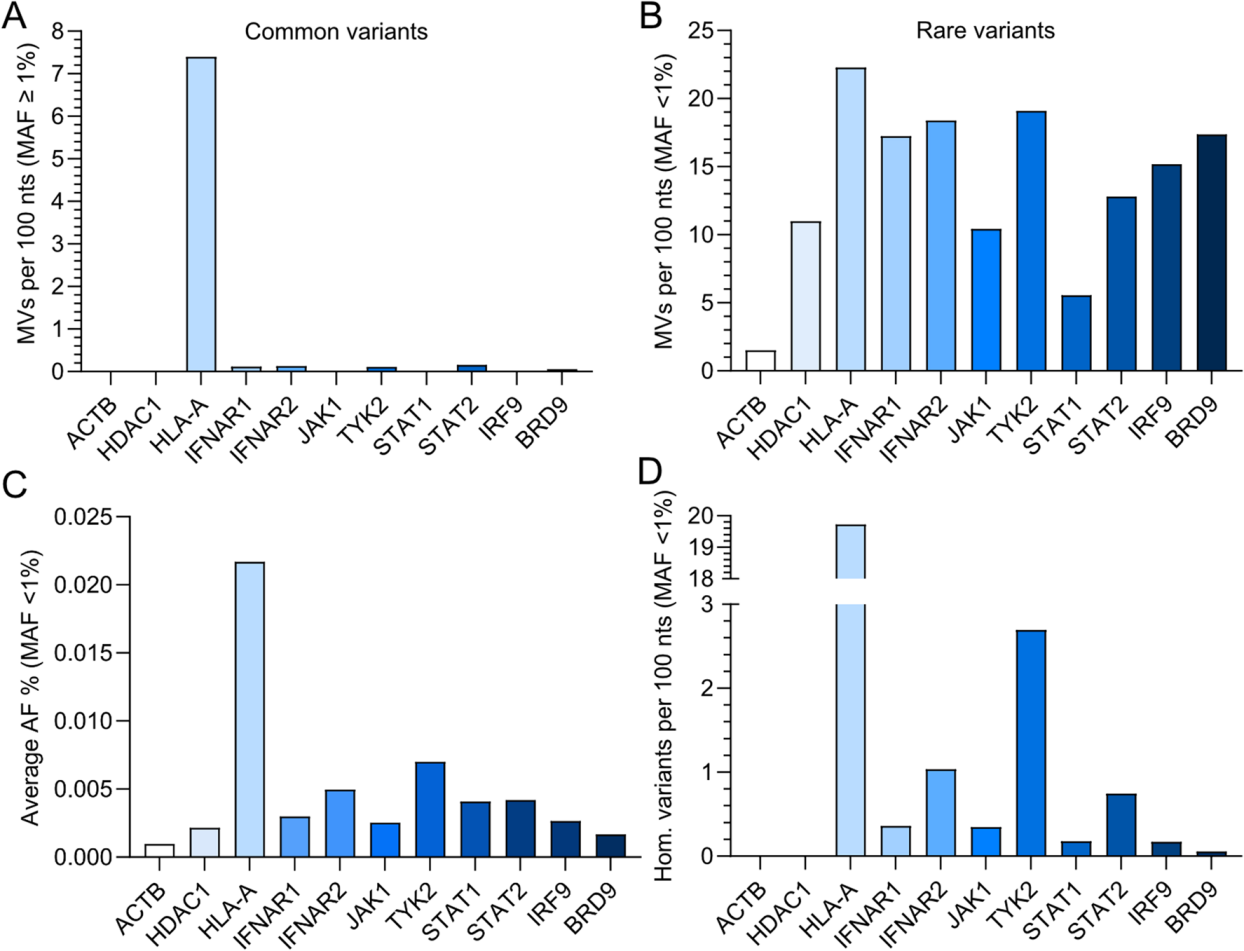
Assessing genetic variation in human IFN signaling pathway components. (**A**) Amount of common missense variants (MVs) calculated per 100 nucleotides (nts) for the indicated human IFN signaling pathway genes as deposited to gnomAD (‘common’ defined as minor allele frequency (MAF) > 1%). The conserved genes *ACTB* and *HDAC1*, as well as the highly variable gene *HLA-A*, serve as reference controls. (**B**) Amount of rare missense variants (MVs) calculated per 100 nucleotides (nts) for the indicated human IFN signaling pathway genes as deposited to gnomAD (‘rare’ defined as MAF < 1%). The conserved genes *ACTB* and *HDAC1*, as well as the highly variable gene *HLA-A*, serve as reference controls. (**C**) Percentage of the mean allele frequency (AF) at which rare genetic alleles of the indicated genes are present in the gnomAD database (MAF < 1%). (**D**) The number of rare variants found in a homozygous manner as calculated per 100 nts for each of the indicated genes (MAF < 1%).

Following a similar strategy to others^19^, we analyzed all missense variants (MVs) in human *BRD9* that are contained in the gnomAD database. Thus, we compared the computed variability of *BRD9* to the computed variabilities of canonical IFN signaling pathway component genes (*IFNAR1*, *IFNAR2*, *JAK1*, *TYK2*, *STAT1*, *STAT2* and *IRF9*), known highly conserved genes (*ACTB* and *HDAC1*), and a known polymorphic gene (*HLA-A*). To account for the varying lengths of each gene, we calculated the number of missense variants per 100 nucleotides (nts). Using this strategy, we confirmed that *HLA-A* is highly variable, with 7.4 common missense variants per 100 nts (‘common’ defined here as minor allele frequency, MAF, ≥ 1%). In contrast, all other tested genes were relatively highly-conserved with respect to common missense variants, with less than 0.2 common missense variants per 100 nts for *IFNAR1*, *IFNAR2*, *TYK2*, *STAT2* and *BRD9*, and no common variants for *ACTB*, *HDAC1*, *JAK1*, *STAT1* and *IRF9* (Fig. 2A). In contrast, rare missense variants (‘rare’ defined here as MAF < 1%) were similar in abundance between most genes (10–20 rare missense variants per 100 nts), with only *STAT1* (around 5 rare missense variants per 100 nts) and *ACTB* (around 1 rare missense variant per 100 nts) having noticeably lower relative numbers of rare missense variants (Fig. 2B). We also calculated the average allele frequency (AF) for rare missense variants to estimate the number of individuals that carry a rare variant, and consequently found that *BRD9* rare missense variants are the second least prevalent after *ACTB* rare variants (Fig. 2C). In line with this, a very low number of individuals were homozygous for *BRD9* rare missense variants (only one described in the canonical transcript: rs376958215), which is similar to highly conserved *ACTB* and *HDAC1*, for which no homozygous rare missense variant carriers have been described on gnomAD (Fig. 2D). These analyses suggest that human *BRD9* is well conserved, and may indicate that rare *BRD9* missense variants are not readily tolerated in humans. This would be consistent with the critical role of BRD9 in the gene expression regulatory functions of the ncBAF chromatin remodeling complex^20^, and potentially also the antiviral IFN system^16–18^.

### Evaluating the impact of rare human *BRD9* missense variants on IFN-stimulated antiviral activity

**Figure 3:**
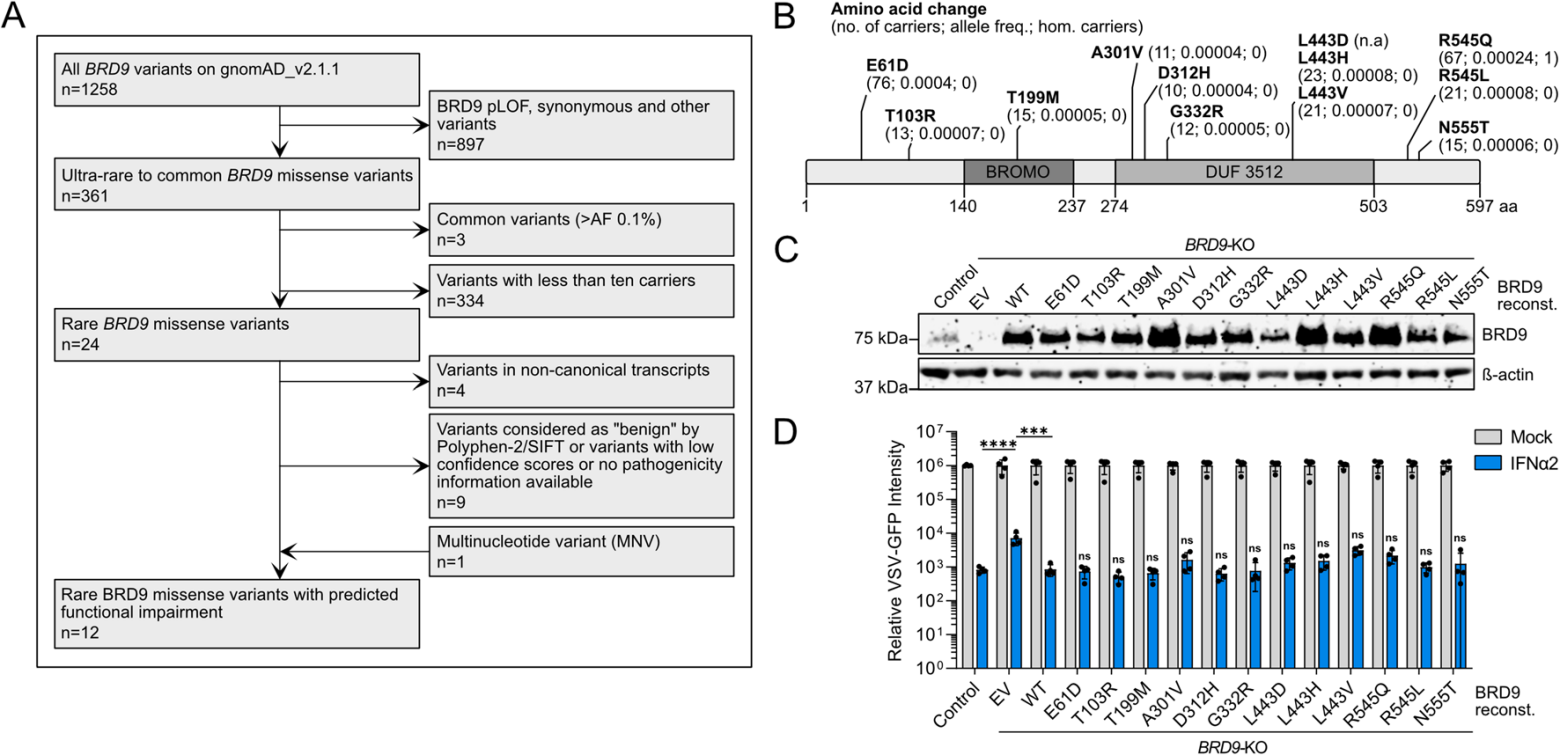
Functional assessment of rare human *BRD9* missense variants on IFN-stimulated antiviral activity. (**A**) Flow diagram mapping out the filtering pipeline used to determine rare human *BRD9* missense variants for functional study. (**B**) Schematic representation of the BRD9 domain structure, with the position of missense variants highlighted. Information in brackets indicates total number of carriers, allele frequency, and total number of homozygotes. (**C**) Western blot analysis of lysates from the non-edited control A549 cell-line and the *BRD9* knock-out (KO) A549 cell-line stably-transduced with lentiviruses expressing each BRD9 variant, wild-type (WT) BRD9, or empty vector control (EV). BRD9 and β-actin were detected with specific antibodies. (**D**) Cells described in (**C**) were stimulated with 0 or 250 IU/mL of IFNα2 for 16 h prior to infection with VSV-GFP at an MOI of 1 PFU/cell. GFP intensity signal measurements taken following infection are plotted. Data represent means and standard deviations from four biologically independent experiments. Individual datapoints are shown. Statistical significance was determined using a two-tailed t-test of the single time-point transformed values of IFNα2-treated conditions (control vs. EV, ****p = 0.0001; EV vs. WT, ***p = 0.0002; WT vs. each indicated BRD9 variant, ns = not significant). Source data underlying the western blots in this figure can be found in the Supplementary Information file.

Genetic variants that damage the host, for example by compromising the ability to protect against severe life-threatening infections, will be subjected to purifying selection and are thus likely to be scarce in human genomes. However, with 1258 human *BRD9* genetic variants reported on gnomAD, we initially decided to focus on rare missense variants that have been described in at least 10 individuals worldwide in order to establish an experimentally feasible, and potentially relevant, set of variants for functional analyses. Our filtering pipeline also excluded variants with synonymous mutations and mutations in splice donor/acceptor sites. Furthermore, we selected rare variants where the missense mutation is predicted to impact subsequent protein functionality according to Polyphen-2 or SIFT (Fig. 3A,B and Dataset S1)^21,22^. The resulting 12 *BRD9* missense variants that we selected for functional testing are distributed across BRD9, and some reside in domains shown to be important for the ability of BRD9 to support IFN-mediated antiviral activity, such as the bromodomain or the DUF3512 domain (Fig. 3B)^16^. Using site-directed mutagenesis we introduced each of these variants individually into a lentivirus-based BRD9 expression construct, which we then used to reconstitute our *BRD9*-KO cell-line, thus generating isogenic cell-lines each stably expressing a different *BRD9* missense variant. As controls, the *BRD9*-KO cell-line was also reconstituted in parallel with a lentivirus vector expressing wild-type *BRD9* (WT; positive-control) or an empty vector (EV; negative-control). Western blot analysis confirmed stable expression of each BRD9 protein in the reconstituted *BRD9*-KO cells, and that levels of each BRD9 missense variant were at least similar to, or higher than, reconstituted wild-type BRD9 (Fig. 3C). To assess the ability of each BRD9 missense variant to support IFN-mediated antiviral activity, we next pretreated cells (or not) with IFNα2 prior to infection with VSV-GFP, and monitored GFP levels over time as a surrogate marker for virus replication. As expected, IFNα2 had reduced antiviral activity in *BRD9*-KO cells as compared to a non-edited control cell-line, and reconstitution of *BRD9*-KO cells with wild-type *BRD9* was largely able to reverse this phenotype. Notably, all *BRD9* missense variants behaved similarly to wild-type *BRD9*, and were able to restore full antiviral activity of IFNα2 in the reconstituted *BRD9*-KO cells (Fig. 3D). These data reveal that these selected rare *BRD9* missense variants, although predicted to be deleterious in nature, do not impair the function of BRD9 in supporting IFN-mediated antiviral activity.

### Evaluating the impact of ultra-rare human *BRD9* loss-of-function variants on IFN-stimulated antiviral activity

**Figure 4:**
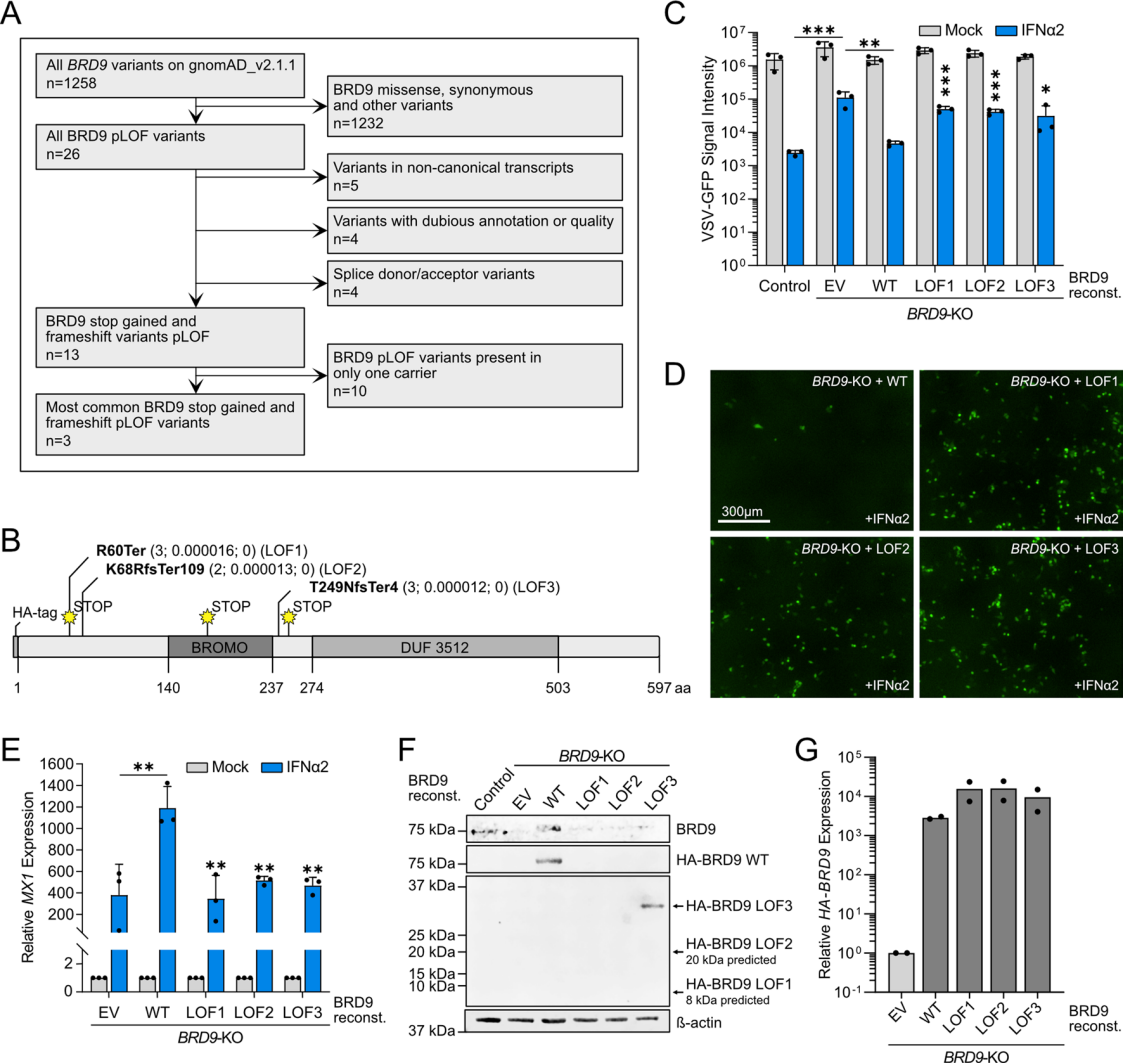
Functional assessment of ultra-rare human *BRD9* loss-of-function (LOF) variants on IFN-stimulated antiviral activity. (**A**) Flow diagram mapping out the filtering pipeline used to determine ultra-rare human *BRD9* LOF variants for functional study. (**B**) Schematic representation of the BRD9 domain structure, with the position of each LOF variant highlighted. Abbreviations: fs, frameshift; Ter, termination. Information in brackets indicates total number of carriers, allele frequency, and total number of homozygotes. (**C**) Non-edited control cells or *BRD9* knock-out (KO) cells stably-transduced with lentiviruses expressing each BRD9 LOF variant, wild-type (WT) BRD9, or empty vector control (EV) were stimulated with 0 or 100 IU/mL of IFNα2 for 16 h prior to infection with VSV-GFP at an MOI of 1 PFU/cell. GFP intensity signal measurements taken following infection are plotted. Data represent means and standard deviations from three biologically independent experiments. Individual datapoints are shown. Statistical significance was determined for IFNα2-treated conditions using unpaired one-tailed t-tests on transformed specific time points for each LOF variant (control vs. EV, ***p = 0.0006; EV vs. WT, **p = 0.0017; WT vs. LOF1, ***p = 0.0002; WT vs. LOF2, ***p = 0.0002; WT vs. LOF3, *p = 0.0388). (**D**) Representative fluorescent images of IFNα2-treated conditions from the experiment described in (**C**). Scale bar represents 300 μm. (**E**) RT-qPCR analysis of *MX1* mRNA in the cell-lines described in (**C**) with or without pretreatment with 1000 IU/mL IFNα2 for 4 h. Values were normalized to *GAPDH* mRNA levels and are relative to the respective mock-stimulated condition**.** Data represent means and standard deviations from three biologically independent experiments. Statistical significance was determined for IFNα2-treated conditions using an unpaired one-tailed t-test (EV vs. WT, **p = 0.0002; WT vs. LOF1, **p = 0.0039; WT vs. LOF2, **p = 0.0023; WT vs. LOF3, **p = 0.0022). (**F**) Western blot analysis of lysates from unstimulated cells described in (**C**). BRD9, the HA-tag and β-actin were detected with specific antibodies. (**G**) RT-qPCR analysis of *HA-BRD9* mRNA in unstimulated cells described in (**C**). Values were normalized to *GAPDH* mRNA levels and are relative to the control cells**.** Data represent means from two biologically independent experiments. Individual datapoints are shown. Source data underlying the western blots in this figure can be found in the Supplementary Information file.

We next sought to assess the functional impact of much rarer (ultra-rare) human *BRD9* variants that are predicted by the Ensembl Variant Effect Predictor^23^ to be loss-of-function (LOF), defined here as frameshift and premature stop codon variants that are likely to profoundly impact mRNA transcript and/or translated protein levels. Our rationale was that rarer and more deleterious variants may have a greater observable effect on IFN-stimulated antiviral activity as they would be more likely to be subjected to purifying selection if they were damaging in humans. Following a similar filtering pipeline as above, albeit retaining variants also described in 2–10 individuals worldwide (Fig. 4A and Dataset S1), we identified 3 naturally occurring human LOF variants in *BRD9* that result in severely truncated versions of BRD9 and which remove functionally relevant domains, such as the bromodomain or the DUF3512 domain (Fig. 4B)^16^. Importantly, none of these LOF variants have been identified as homozygous in any individual to date. We introduced each of these ultra-rare BRD9 LOF variants individually into the lentivirus-based expression system, cloning in-frame an N-terminal HA-tag to facilitate subsequent detection. We then stably reconstituted the *BRD9*-KO cell-line with each of these HA-tagged BRD9 LOF variants, as well as HA-tagged full-length wild-type BRD9 as a positive control. While IFNα2 had reduced antiviral activity against VSV-GFP in *BRD9*-KO cells as compared to the non-edited control cell-line, reconstitution of *BRD9*-KO cells with HA-tagged full-length wild-type BRD9 reversed this deficit (Fig. 4C). In contrast, individual expression of any of the 3 BRD9 LOF variants was unable to restore the antiviral action of IFNα2 against VSV-GFP (Fig. 4C,D). These virus-based findings were further supported by gene expression analysis of *MX1* as a prototypic ISG, revealing that full-length wild-type BRD9, but not any of the 3 BRD9 LOF variants, permitted *BRD9*-KO cells to respond effectively to IFNα2 (Fig. 4E). Notably, while western blot analysis could confirm stable protein expression of the HA-tagged WT BRD9 and longest BRD9 LOF variant (LOF3), the shorter LOF protein variants, LOF1 and LOF2, could not be detected in the reconstituted cell-lines (Fig. 4F). This failure to express to detectable levels is consistent with the likely instability of such proteins with very premature stop codons (e.g. LOF1) or long stretches of nonsense (LOF2). Indeed, RT-qPCR analysis revealed that the mRNAs encoding HA-tagged WT BRD9 and all 3 BRD9 LOF variants were expressed to similar levels (Fig. 4G). These functional data indicate that severely truncated ultra-rare *BRD9* LOF variants, at least when present homozygously, are likely to render cells less able to mount an IFN-mediated antiviral response.

## Concluding remarks

BRD9 defines the ncBAF chromatin remodeling complex that, together with the canonical STAT1-STAT2-IRF9 transcription factor complex, has previously been identified as an important contributor to efficient IFN-stimulated gene transcription and antiviral immunity^16–18^. Our evaluation of *BRD9* genetic variants has now identified that severely truncated ultra-rare *BRD9* LOF alleles exist in the human population, and revealed that under conditions where only these alleles are present there is compromised ability of IFN to fully protect cells against virus infection. While our data currently cannot be used to draw conclusions about the impact of wild-type/LOF *BRD9* heterozygosity on IFN action, we can at least add *BRD9* as a potential gene where severe functional defects affecting both alleles are likely to impact innate antiviral immunity. Thus, the status of *BRD9* could perhaps be considered in future genetic studies when trying to understand the otherwise unexplained susceptibility of a very rare individual to virus infection. Indeed, there have been no homozygous *BRD9* LOF variant carriers reported on gnomAD, which may suggest negative selection against dysfunctional BRD9, probably due to its essential gene-regulatory roles in innate antiviral immunity or other pathways^16–18,24^. Interestingly, our analyses failed to identify any rare *BRD9* missense variants (present in at least 10 individuals worldwide) that compromised the action of BRD9 in innate immune control, despite all of the tested variants being predicted bioinformatically to be damaging by SIFT and/or PolyPhen-2^21,22^. It is possible that, similar to the LOF variants, any truly damaging variant will be selected against in the general human population, and would thus be ultra-rare, particularly if dominant-negative. Thus, a limitation of the present study is the lack of functional analysis of ultra-rare *BRD9* missense variants, which were omitted in order to maintain experimental feasibility and potential relevance to multiple individuals. However, future efforts to predict and elucidate the impact of *BRD9* missense variants on human susceptibility to infectious diseases should probably prioritize ultra-rare *BRD9* variants, including severely truncated LOF variants.

## Materials and methods

### Cells and interferon

A549 and HEK293T cells (both originally from ATCC) were cultured at 37 °C and 5% CO_2_ in Dulbecco’s Modified Eagle’s Medium (DMEM; Gibco) supplemented with 10% (v/v) fetal calf serum (FCS), 100 U/mL of penicillin and 100 μg/mL of streptomycin (#15,140–122; Gibco). The A549-derived *BRD9*-KO cell-line and non-edited control cell-line (clone pair #3) were generated and characterized by western blot and amplicon-based NGS previously^16^. IFNα2a (Roferon-A; Roche; termed IFNα2 here) was used at the indicated concentrations.

### Plasmids, lentiviruses and cell-lines

cDNA encoding wild-type BRD9 was PCR-amplified from existing vectors^16^ and cloned into pLVX-IRES-Puro (Takara) with or without an in-frame N-terminal HA tag sequence via *EcoRI/NotI* restriction sites. BRD9 variants were subsequently generated by standard overlap-extension PCR using Q5 Hot Start High-Fidelity DNA polymerase (NEB) and cloned in a similar manner. The inserts of all new constructs were Sanger-sequenced to confirm identities of the respective variant. To generate polyclonal cell lines expressing the gene of interest, lentiviral stocks were prepared by co-transfecting HEK293T cells with each pLVX-IRES-Puro-based plasmid, together with pCMV-VSV-G and psPAX2 as done previously^16^. Lentiviral supernatants were harvested 24 and 48 h post-transfection, filtered through a 0.45 μm filter and stored at − 80 °C. The A549-derived *BRD9*-KO cell-line or non-edited A549 control cell-line were then transduced with the indicated lentivirus stock for 48 h in the presence of 8 μg/ml of polybrene (Millipore) prior to stable selection with puromycin.

### SDS-PAGE and western blotting

Cell lysates were prepared in 2X urea disruption buffer (6 M urea, 4% SDS, 1 M β-mercaptoethanol, bromophenol blue), sonicated, and heated to 98 °C for 2 min. Proteins were separated by SDS-PAGE on 4–12% NuPAGE Bis–Tris gradient gels (Life Technologies) and transferred to 0.45 µm nitrocellulose membranes (GE Healthcare, Amersham). The indicated proteins were detected by standard methods using antibodies specific for β-actin (mouse, beta-actin (C4), sc-47778, Santa Cruz), BRD9 (rabbit, A303-781A, Bethyl Laboratories, Inc.), or the HA-tag (mouse, HA-Tag (6E2), #2367S, Cell Signaling Technology). The secondary antibodies used were IRDye 800CW goat anti-mouse IgG (#926–32,211, Li-Cor) and IRDye 680RD goat anti-rabbit IgG (#926–68,071, Li-Cor). A Li-Cor Odyssey scanner was used for detection. Source data underlying all the western blots can be found in the Supplementary Information file.

### Infection assays

Approximately 2.5 × 10^4^ cells per well were seeded into 96-well plates. Twenty-four hours later, cells were stimulated with the indicated amounts of IFNα2 for 16 h prior to infection with a recombinant GFP-expressing vesicular stomatitis virus (VSV-GFP; kind gift of Peter Palese, Icahn School of Medicine at Mount Sinai, USA) at an MOI of 1 PFU/mL. Infections were monitored in live cells via GFP expression using the IncuCyte ZOOM Live-Cell analysis system (Sartorius) for the times indicated. The amount of infected cells, total Green Integrated Intensity (Green Calibrated Unit × µm^2^/image) values, or raw images, were exported at the indicated time points.

### RT-qPCR analyses

Total cellular RNA was extracted using the ReliaPrep RNA Cell Miniprep System (Promega) according to the manufacturer’s instructions. cDNA was synthesized from 300–600 ng of total RNA using Superscript III reverse transcriptase (ThermoFisher) and an Oligo(dT)_15_ primer (Promega). RT-qPCR was performed on a 7300 Real-Time PCR system (Applied Biosystems) using Fast EvaGreen qPCR Master Mix Kit (Biotium) and primers for *MX1*: Hs.PT.58.26787898 (Integrated DNA Technologies); *HA-BRD9* (5′-TGTACCCATACGATGTTCCAGAT-3′ and 5′-AGGGGCTTGTCGGCATAATC-3′) and *GAPDH* (5′-CTGGCGTCTTCACCACCATGG-3′ and 5′-CATCACGCCACAGTTTCCCGG-3′). The delta-delta-cycle threshold (∆∆Ct) was determined relative to control samples. *MX1* or *HA-BRD9* gene expression was normalized to *GAPDH* expression.

### Bioinformatic analysis of human genetic variants in *BRD9*

Human genetic variant information was obtained using the publicly available database gnomAD_v2.1.1 (https://gnomad.broadinstitute.org/), accessed on 22.10.2019. In brief, after downloading all missense *BRD9* variants, several filters (as described in the figures) were applied to exclude/include LOF, synonymous and other variants (i.e. variants in the 3’UTR, intron and splice regions), too common/rare variants, as well as variants in non-canonical transcripts or variants with estimated ‘benign’ pathogenicity scores (variants estimated to be ‘probably damaging’ or ‘possibly damaging’ according to Polyphen-2 and/or ‘deleterious’ according to SIFT were included). Of note, one BRD9 locus (amino acid 443) was found to be a multi-nucleotide variant, which is defined as two or more variants located in close proximity on the same haplotype in an individual. The variants both occur within the same codon (5-876,271-A-T, rs769713688 and 5-876,272-G-C, rs772994298) and produce a third variant when combined (5–876,271-AG-TC, no reference SNP (rs) number allocated). Since the accumulated impact of two variants combined in one transcript may have distinct functional consequences compared to the respective individual variants alone, this combined variant was included in our analysis.

### Statistical Analyses

Statistical analyses were performed using GraphPad Prism 9.2.0 (GraphPad Software, San Diego, California USA). Figure 3D was analyzed using an unpaired two-tailed t-test of the transformed values of single time points (30 hpi for experiment 1 and 59 hpi for experiments 2–4). For display, data were normalized to 10^6^. Since we anticipated the *BRD9* LOF variants to have a similar effect on virus replication as *BRD9* KO, we analyzed the VSV-GFP assays (Fig. 4C) using unpaired one-tailed t-tests on transformed specific time points. To test for differences in *MX1* expression (Fig. 4E), an unpaired one-tailed t-test was performed on the observed values relative to mock. *p* values for significance are indicated in the figure legends.

## Supplementary Information

Supplementary Information 1.

Supplementary Information 2.

## Data Availability

All data generated or analysed during this study are included in this article [and its supplementary information files].
