# Supplementary figures and images for: Ultra-Rare BRD9 Loss-of-Function Variants Limit the Antiviral Action of Interferon
Source: Sci Rep. 2022 Sep 13;12:15360. doi: 10.1038/s41598-022-19648-w (PMC9468519; doi:10.1038/s41598-022-19648-w)

# Supplementary Information - western blot source data for Figures 1, 3 and 4.

## Figure 1

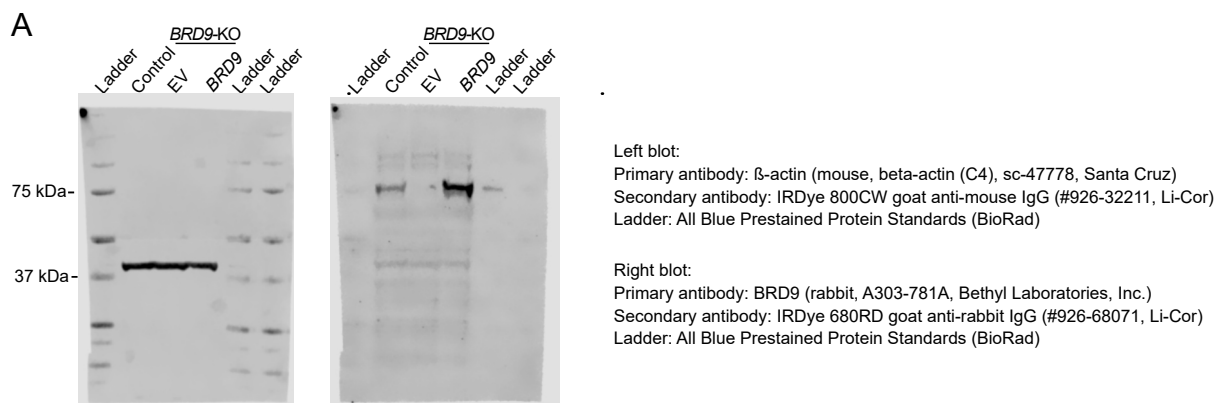

## Figure 3

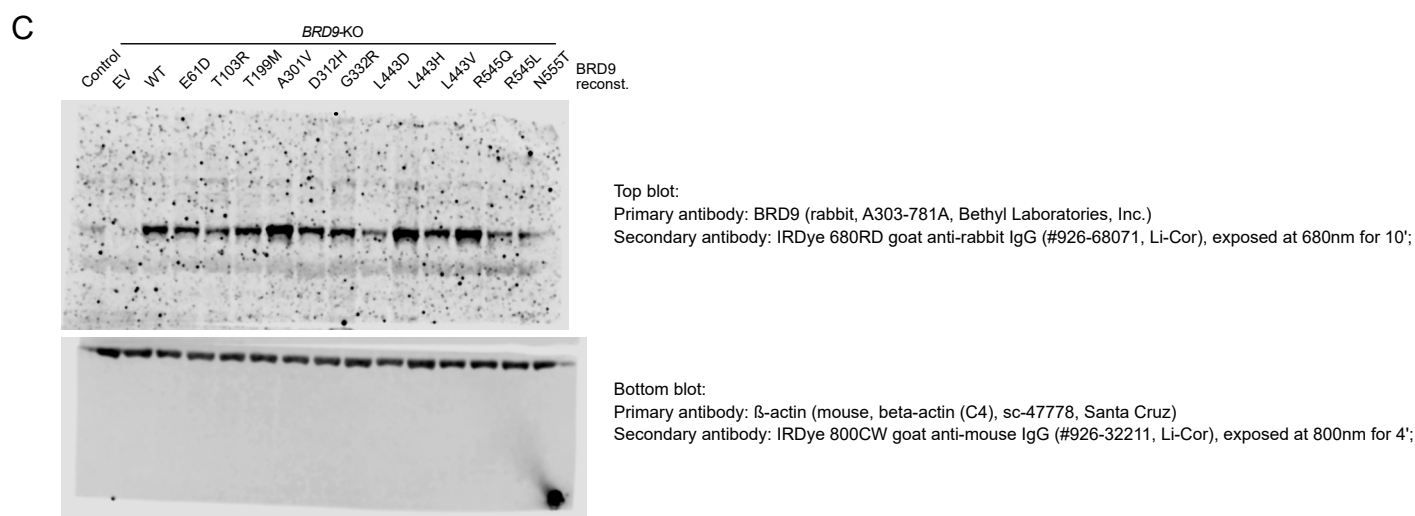

## Figure 4

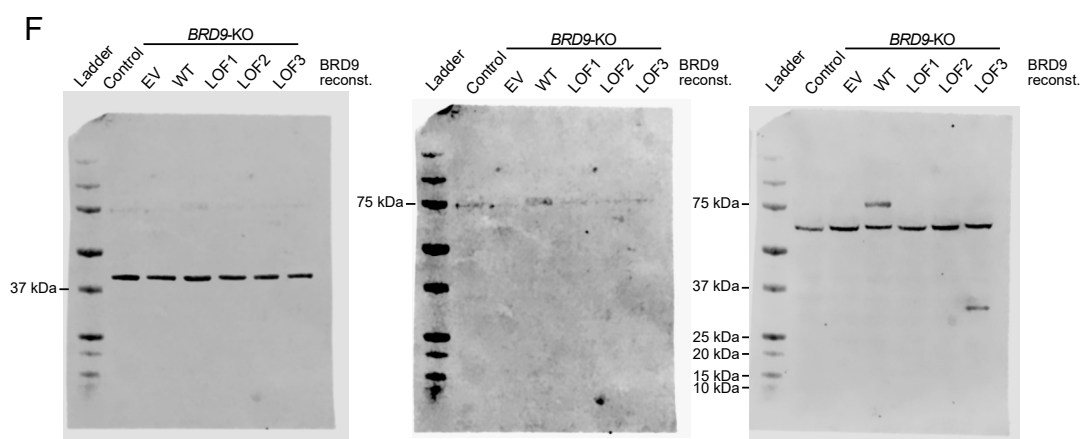

Supplement: Supplementary file 2 — Supplementary Information 2. [file 41598_2022_19648_MOESM2_ESM.pdf]
